# Development and verification of the nomogram for dilated cardiomyopathy gene diagnosis

**DOI:** 10.1038/s41598-022-13135-y

**Published:** 2022-05-26

**Authors:** Li-qiang Zhou, Chuan Liu, Yeqing Zou, Zhi-qing Chen

**Affiliations:** Jiangxi Key Laboratory of Molecular Medicine, The Second Affiliated Hospital of Nanchang University, 1 Minde Road, Donghu District, Nanchang, 330006 Jiangxi China

**Keywords:** Biomarkers, Diagnostic markers, Predictive markers, Cardiovascular biology

## Abstract

Dilated cardiomyopathy (DCM) is a primary myocardial disease of unclear mechanism and poor prevention. The purpose of this study is to explore the potential molecular mechanisms and targets of DCM via bioinformatics methods and try to diagnose and prevent disease progression early. We screened 333 genes differentially expressed between DCM and normal heart samples from GSE141910, and further used Weighted correlation network analysis to identify 197 DCM-related genes. By identifying the key modules in the protein–protein interaction network and Least Absolute Shrinkage and Selection Operator regression analysis, seven hub DCM genes (CX3CR1, AGTR2, ADORA3, CXCL10, CXCL11, CXCL9, SAA1) were identified. Calculating the area under the receiver’s operating curve revealed that these 7 genes have an excellent ability to diagnose and predict DCM. Based on this, we built a logistic regression model and drew a nomogram. The calibration curve showed that the actual incidence is basically the same as the predicted incidence; while the C-index values of the nomogram and the four external validation data sets are 0.95, 0.90, 0.96, and 0.737, respectively, showing excellent diagnostic and predictive ability; while the decision curve indicated the wide applicability of the nomogram is helpful for clinicians to make accurate decisions.

## Introduction

Dilated cardiomyopathy (DCM) is a primary cardiomyopathy characterized by left ventricular dilation and systolic insufficiency, accompanied by heart remodeling and fibrosis^1^. DCM is usually progressive aggravation and gradually developed into severe congestive heart failure, which will seriously threaten the survival rate of patients^2^. Currently, heart transplantation is the most effective treatment for dilated cardiomyopathy with long-term heart failure that has failed medical treatment, and the prognosis is greatly improved^3^. Early diagnose and intervention of DCM are particularly important for improving the prognosis and quality life of DCM patients.

DCM is a genetic disease involving post-inflammatory or genetic causes. The genetic characteristics of DCM can be accurately diagnosed in advance, and preventive measures can be taken in time to avoid the further occurrence and development of the disease^4^. Elizabeth et al. re-evaluated a number of genes that are important in the progression of DCM, which has implications for the diagnosis and treatment of DCM^5^. And it has become feasible to use the second-generation sequencing to study the genetics of DCM, which deeply understand the molecular biological mechanism of DCM and find the biological targets for the diagnosis and treatment of DCM.

In this study, we conducted an in-depth analysis of DCM samples and normal samples in the GSE141910 data set through a variety of statistical methods, obtained differentially expressed genes that were significantly correlated with DCM, and studied the underlying mechanism of these genes' impression of DCM. Furthermore, we obtained the biomarkers of DCM and analyzed its diagnostic predictive value. Based on this, we constructed a genetic diagnosis model of DCM, and through multiple methods of testing, the results showed that it has excellent predictive sensitivity. This research can help clinicians diagnose DCM early and has far-reaching significance for the prevention and treatment of DCM.

## Materials and methods

### Statement

All methods were carried out in accordance with relevant guidelines and regulations.

### Data collection and processing

We obtained the dataset GSE141910 from the Gene Expression Omnibus (GEO) database (http://www.ncbi.nlm.nih.gov/geo/), this dataset contained 166 samples are surgical myectomy tissues from patients with dilated cardiomyopathy (DCM), and 166 samples are patients without heart failure (NF) as control cardiac tissues. Subsequently, we implemented the eBayes method of the "limma" package with R 3.6.1 software^6^. Simultaneously, we set |log_2_ fold change (FC)|  > 1.5 and false discovery rate (FDR) < 0.05 as the filter condition to identify Differentially expressed genes (DEGs) between the DCM and NF samples.

### Weighted gene co-expression network analysis (WGCNA)

We used the “WGCNA” package to performed a network construction^7^. WGCNA is an analysis method that analyzes gene expression patterns in multiple samples. It can cluster genes with similar expression patterns and analyze the association between modules and specific traits or phenotypes. WGCNA started at the level of DEGs, dividing them into several modules based on their patterns of connection with other genes, thus identifying the module most relevant to DCM. An unsupervised co-expression relationship was initially built on the basis of the adjacency matrix of connection strengths by using Pearson’s correlation coefficients for gene pairs. After selecting the appropriate power value, the adjacency matrix of the gene expression data of GC patients is clustered using topological overlap matrix analysis. Finally, the dynamic tree cut algorithm was applied to the dendrogram for module identification with the mini-size of module gene numbers set as 50 and a cut height of 0.85. We select the genes in the modules with the highest correlation with DCM for further analysis.

### Gene function enrichment analysis

In order to study the functions of genes in the key module, we use the “ClusterProfiler” package to perform gene ontology (GO) and Kyoto encyclopedia of genes and genomes (KEGG) enrichment analysis on these genes^8^. We used Database for Annotation, Visualization and Integrated discovery GO function is carefully evaluated from three aspects: biological process (BP), molecular function (MF) and cellular component (CC). KEGG is helpful for studying an integrated network of gene and expression information, systematically analyzing the metabolic pathways of gene products in cells and a database of the functions of these gene products. The enrichment results with *P*-value and FDR value below 0.05 are considered meaningful. The visual GO and KEGG enrichment results were performed by “GOplot” R packages^9^.

### Construction protein protein interaction (PPI) network

STRING is a database for searching known and predicted protein interactions (Version 10.5; http://stringdb.org/)^10^. We used this database to build a PPI network to mine the core regulatory genes of DCM. We uploaded the gene of the key module to the STRING database to get a PPI play. Then, we used Cytoscape software (version: 3.7.2, http://www.cytoscape.org/) to visualize the PPI and perform further analysis^11^. Cytoscape's network analysis analyzes the degree value of all nodes. All nodes are arranged in order from the inside to the outside according to the degree size, and the nodes with degree > 10 are selected. Cytoscape’s pluggable unit molecular complex detection (MCODE) is a module for screening PPI networks^12^. Take the cut-off degree = 2, cut-off point = 0.2, *k*-core = 2, max depth = 100 as the network score and cluster search parameter settings, then analyze the hub modules. If there is a gene with degree > 10 and exists in the hub module, it is regarded as a candidate gene, and further analysis is performed.

### LASSO regression to identify the hub gene

The least absolute shrinkage and selection operator (LASSO) method, which is suitable for the reduction of high-dimensional data, was used to select the most useful predictive features from the primary data set in this study. Using the LASSO regression model, among the candidate genes, all candidate genes are reduced to a limited potential predictor. If the penalization coefficient lambda (*λ*) is large, there is no effect on the estimated regression parameters, but as the *λ* gets smaller, some coefficients may be shrunk toward zero. We use “glmnet” package to screen the hub genes that predict DCM from these candidate genes^13^. And use the "ROC" package to analyze the ROC values of these hub genes and evaluate the sensitivity of their predictions.

### Construction and verification of DCM diagnostic signature and nomogram

We used the "rms" package to perform multiple logistic regression on the obtained central genes and obtained the corresponding probability (OR) and regression coefficient (*β*). In order to construct a diagnostic model for predicting DCM, according to the formula: Risk score = *β*1 * EXP1 + *β*2 * EXP2 +$\cdots$ + *β*i * EXPi, thereby predicting the incidence of DCM. We used a nomogram to visualize the DCM prediction model, assigned a corresponding score to each gene expression, and then added the scores of all hub genes to get the total score. By drawing a vertical line at the total score obtained, the risk value of DCM can be clarified. In order to quantify the predictive performance of the nomogram, a calibration curve was drawn to evaluate the consistency between the prediction and observation of the nomogram, and the calibration of the model was described according to the consistency between the predicted risk and the actual result. Then, the consistency index (C-index) was calculated in GSE116250^14^, GSE43435^15^, GSE21610^16^ to verify the prediction performance. In addition, we used the "rmda" package to draw a decision curve (DCA) to calculate the net benefit of diagnostic signatures and evaluate diagnostic performance.

### Consent for publication

All authors agree to the publication of this article.

## Results

### Identify DEGs of DCM

**Figure 1:**
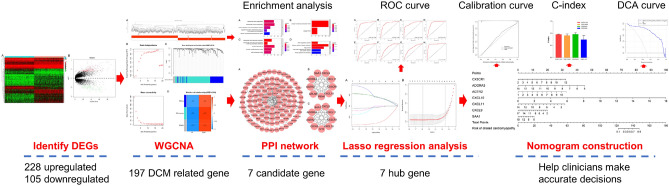
Flow chart of this research.

**Figure 2:**
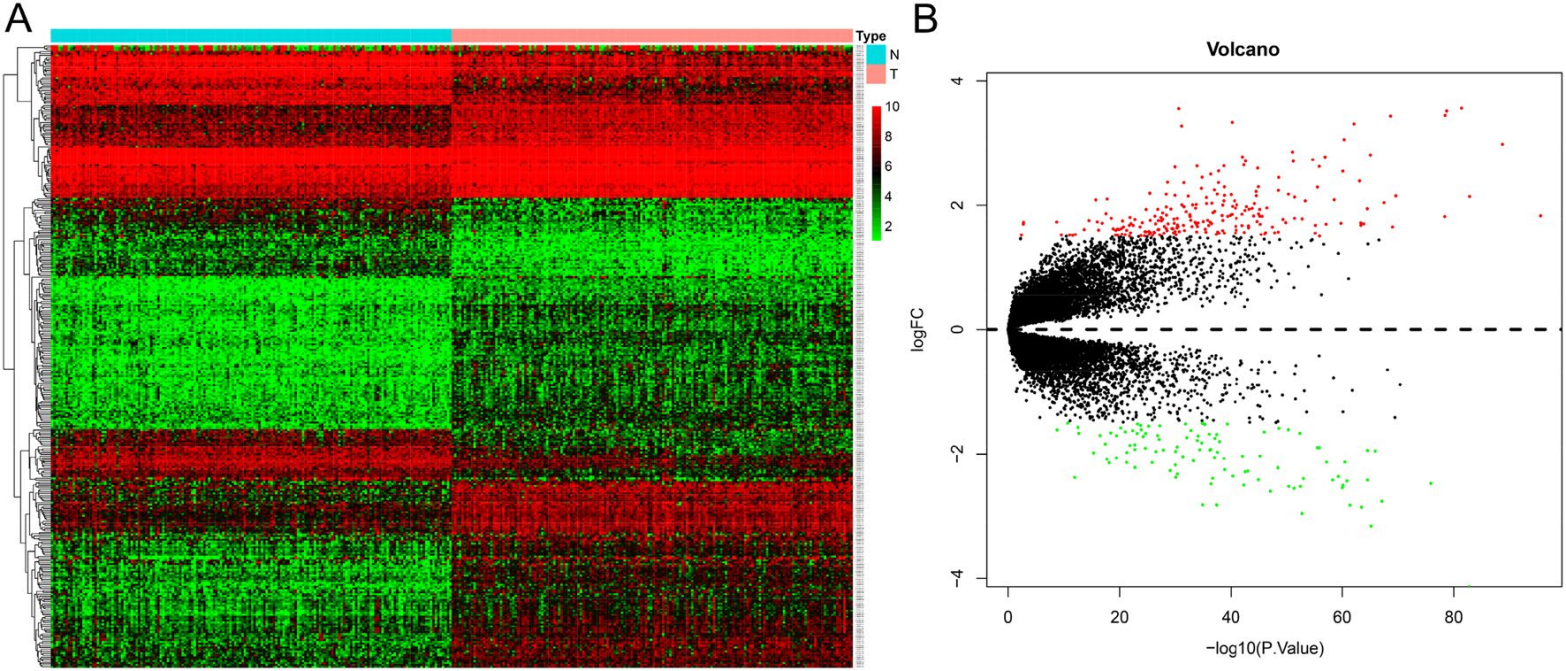
Identify the 333 differential genes of dilated cardiomyopathy. (**A**) Heat map. (**B**) Volcano plot.

This study analyzed the hub genes of DCM and its underlying mechanisms via various statistical methods and analyzed its ability to predict DCM. The entire workflow is shown in Fig. 1. We identified 333 differentially expressed genes in 166 NF and 166 DCM samples that met the screening criteria (Supplementary Table 1). A heat map was aimed for assessing the levels of the DEGs expression to distinguish the DCM and control samples (Fig. 2B). Among these DEGs, 228 were upregulated and 105 were downregulated (Fig. 2A).

### WGCNA identification of key modules

**Figure 3:**
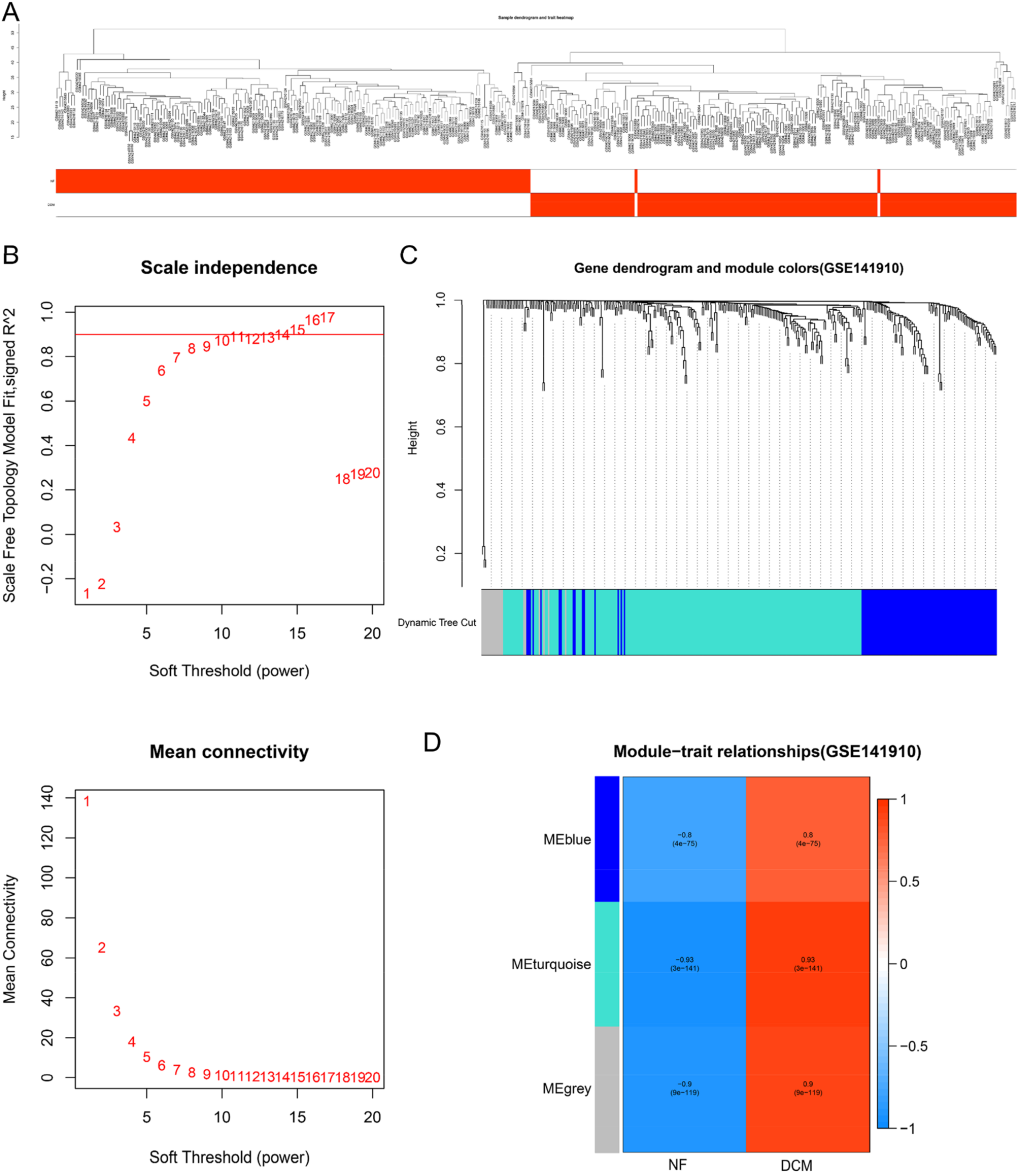
WGCNA identified 197 genes closely related to dilated cardiomyopathy. (**A**) The distribution of different samples. (**B**) Analyze the best Power value. (**C**) Dendrogram of all clusters expressing genes based on the difference metric (1-TOM). (**D**) The heat map shows the correlation between each module and dilated cardiomyopathy.

We analyzed whether there are outliers in each sample, and then performed hierarchical clustering. The results suggested that there are no samples with outliers, and the dendrogram contains all 332 samples (Fig. 3A). In WGCNA analysis, we selected the soft-thresholding power to identify the relatively balanced scale independence and mean connectivity. As shown in Fig. 3B, under the condition that the cutting height is set to 0.85, power = 15 could be as the power value of soft-threshold. Then, a total of three modules were generated by average linkage hierarchical clustering based on the 333 input genes (Fig. 3C). Of which, after calculating the MS of each module-trait correlation by WGCNA, the three modules related to DCM are MEblue module (*r* = 0.80, *P* = 4.00E−75), MEurquoise module (*r* = 0.93, *P* = 3.00E−141), MEgrey module (*r* = 0.90, *P* = 9.00E−119). The MEturquoise module had the highest correlation, and it contained 197 genes that could potentially be associated with DCM (Fig. 3D).

### GO and KEGG function analysis

**Figure 4:**
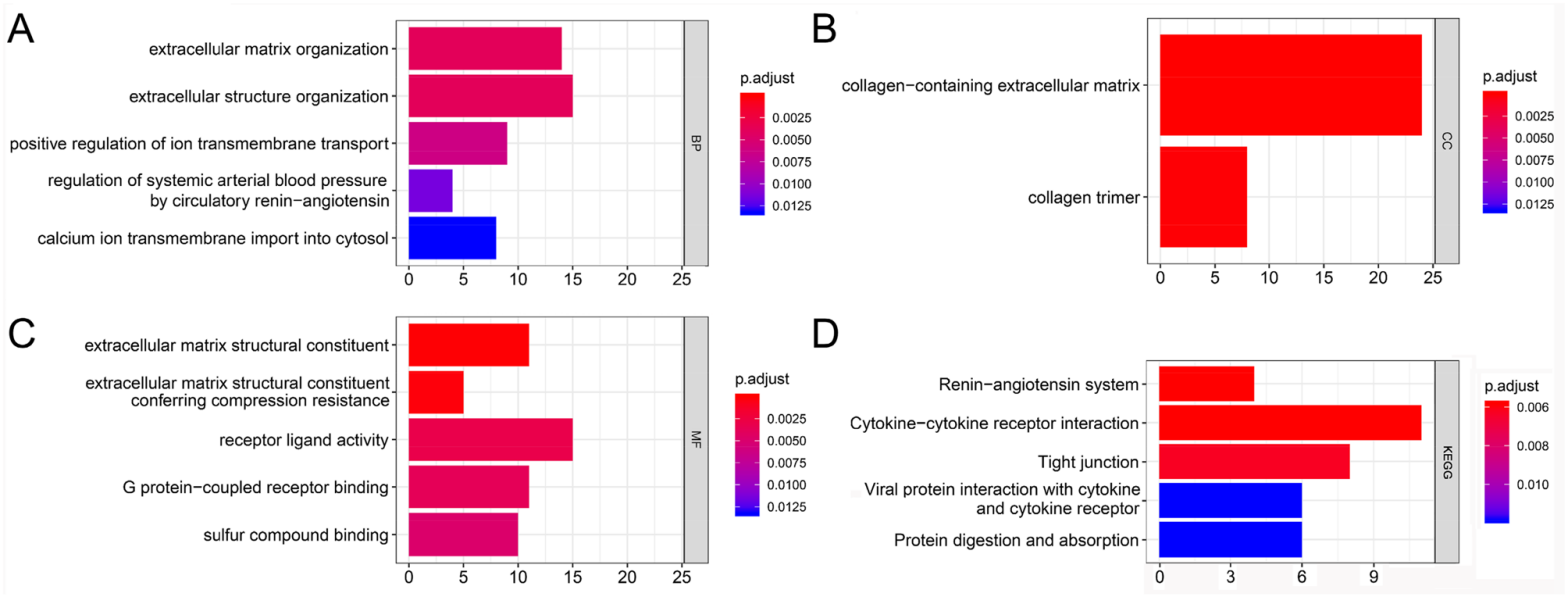
Gene function enrichment analysis. (**A**) Biological process. (**B**) Cellular components. (**C**) Molecular function. (**D**) KEGG.

GO and KEGG functional enrichment analysis to understand the potential mechanism of genes in the MEturquoise module (Fig. 3D) affecting DCM, we found the enrichment results of Top5. There are 93 items in GO enrichment analysis. The biological processes are enriched in 82 items such as extracellular matrix organization, extracellular structure organization, positive regulation of ion transmembrane transport, regulation of systemic arterial blood pressure by circulatory renin–angiotensin, calcium ion transmembrane import into cytosol, etc. Entry (Fig. 4A); Cellular components are enriched in collagen-containing extracellular matrix, collagen trimer (Fig. 4B); molecular functions are enriched in extracellular matrix structural constituent, extracellular matrix structural constituent conferring compression resistance, receptor ligand activity, G protein-coupled receptor binding, sulfur compound binding, lipopeptide 8 items such as binding (Fig. 4C). KEGG results suggested that these genes may affect DCM through renin–angiotensin system, cytokine–cytokine receptor interaction, Tight junction, Viral protein interaction with cytokine and cytokine receptor, Protein digestion and absorption (Fig. 4D).

### PPI network screening candidate genes

**Figure 5:**
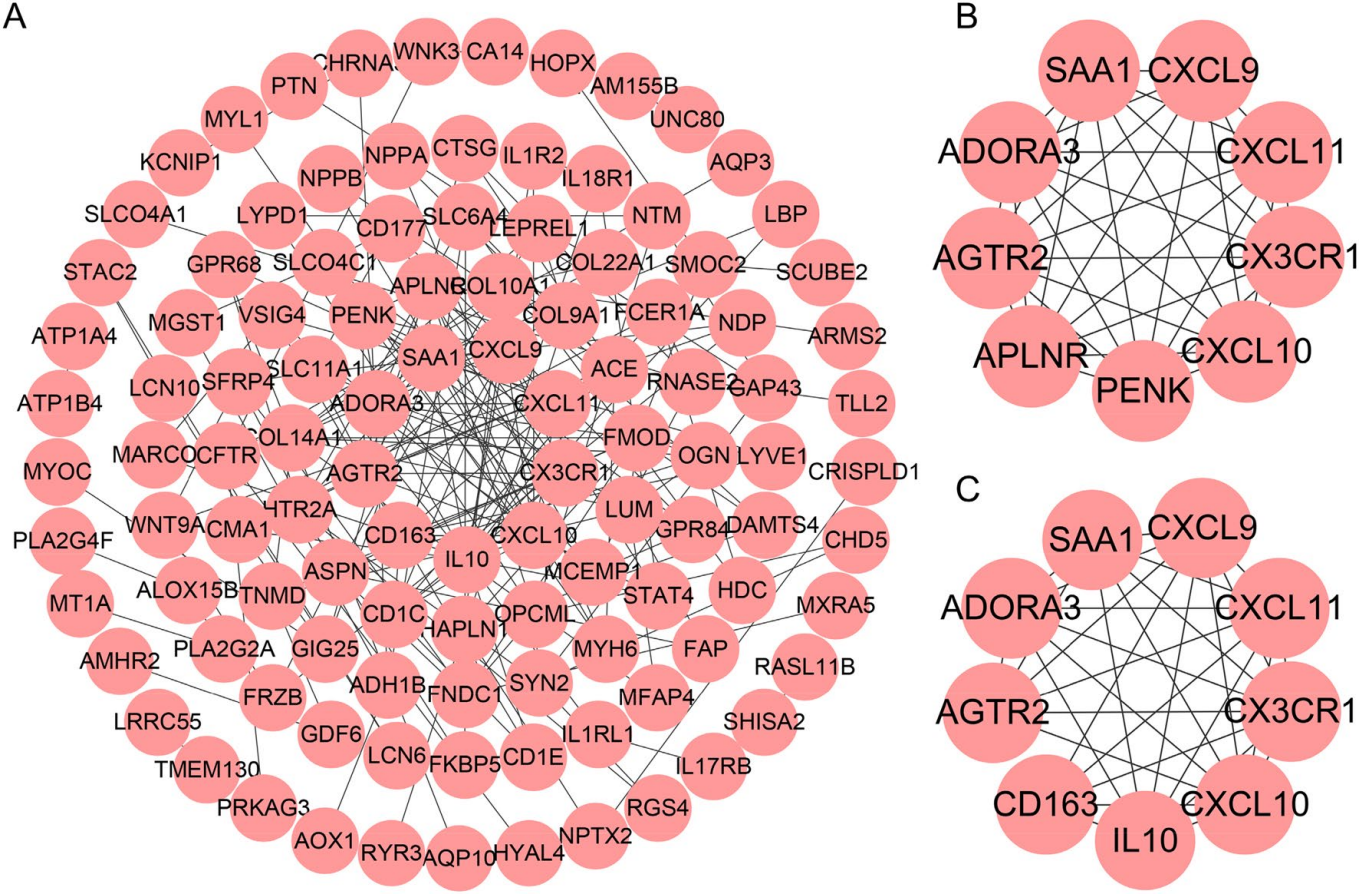
PPI network screening 7 candidate genes. (**A**) A sub-network composed of genes whose degree is greater than 10. (**B**) The key module identified by MCODE.

We used String to form a PPI network with 109 nodes and 206 edges from 197 genes in the turquoise module (Fig. 5A). And we analyzed the degree value of each node and selected 9 genes greater than 10 to form a sub-network with 9 nodes and 36 edges (Fig. 5B). Next, we analyzed the key modules of the entire PPI network using MCODE, and obtained a network composed of 9 genes (Fig. 5C). The genes with degree > 10 were consistent with the genes of the key modules, and they were used for further analysis. What is interesting is that the degree > 10 genes are the same as 7 genes in the key modules, including CXCR1, AGTR2, ADORA3, CXCL10, CXCL11, CXCL9, SAA1. We used these genes for further analysis to screen for genes that best predicted DCM.

### Construction DCM diagnosis model

**Figure 6:**
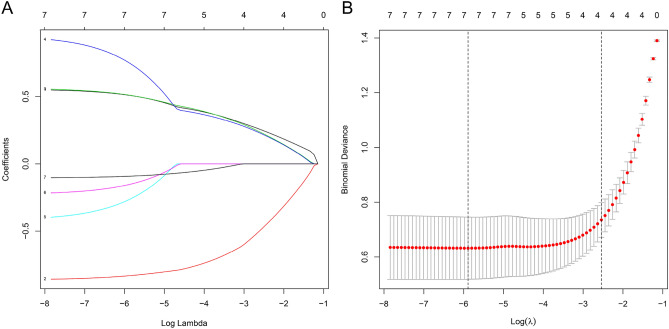
Lasso regression removes collinearity to identify 7 hub genes.

**Figure 7:**
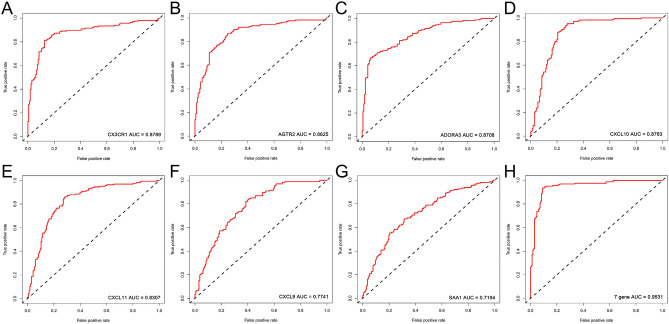
Subject's operable curve analysis 7 hub genes individually and jointly predict the ability of dilated cardiomyopathy. (**A**) CX3CR1. (**B**) AGTR2. (**C**) ADORA3. (**D**) CXCL10. (**E**) CXCL11. (**F**) CXCL9. (**G**) SAA1. (**H**) Combine 7 genes.

Lasso regression is used to screen genes that predict DCM. We set the value of *λ* to 2.09E−03, it indicated that the regression coefficients of most variables in the model tend to be stable, and the results showed that the above 7 genes are all predictors of DCM (Fig. 6). To this end, we drew the receiver operating characteristic curve (ROC) of these 7 genes, and calculated the area under the curve (AUC). The AUC value of each gene (Fig. 7A–G): CXCR1 (AUC = 0.88), AGTR2 (AUC = 0.86), ADORA3 (AUC = 0.87), CXCL10 (AUC = 0.88), CXCL11 (AUC = 0.83), CXCL9 (AUC = 0.77), SAA1 (AUC = 0.72). We found that the AUC value after integration of 7 genes is 0.95 (Fig. 7H), which has the best sensitivity compared to individual genes. Based on these 7 hub genes, we used a multiple logistic regression to construct a predictive model to predict the risk of DCM (Table 1).

**Table 1:** Multiple logistic regression of dilated cardiomyopathy.

Gene ID	Regression coefficients (*β*)	Odds ratio (OR)	95% Confidence interval (CI)	*P*-value
CX3CR1	0.56	1.74	1.25–2.47	1.35E−03
ADORA3	− 0.86	0.42	0.28–0.61	1.78E−05
AGTR2	0.56	1.76	1.31–2.42	2.89E−04
CXCL10	0.96	2.60	1.66–4.31	8.35E−05
CXCL11	− 0.42	0.65	0.53–0.98	4.43E−02
CXCL9	− 0.23	0.80	0.58–1.09	1.59E−01
SAA1	− 0.10	0.90	0.79–1.03	1.25E−01

### DCM nomogram establishment and verification

**Figure 8:**
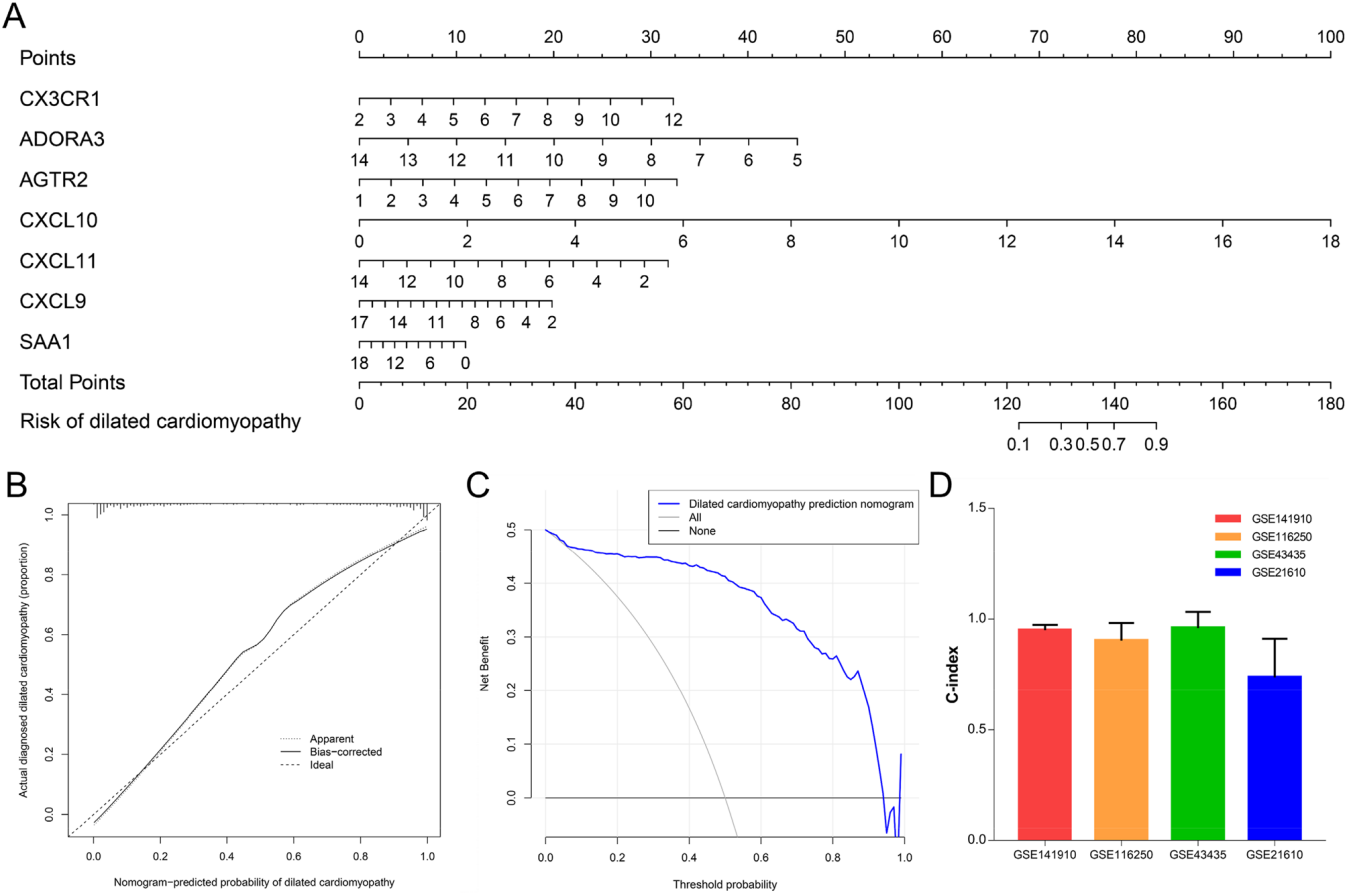
The establishment and verification of the diagnosis and prediction model of dilated cardiomyopathy. (**A**) 7 Predictive nomograms of genetic diagnosis. (**B**) Calibration curve. (**C**) Decision curve. (**D**) 4 C-index of external validation data set.

In order to understand the relationship between each gene and the onset of DCM more intuitively, we constructed a nomogram based on multiple logistic regression (Fig. 8A). By detecting the expression of each hub gene, the corresponding score was obtained, and the incidence of DCM can be judged according to the total score obtained. In order to verify the accuracy of the nomogram prediction, we draw a Calibration curve. According to Fig. 8B, the predicted curve is almost the ideal curve. In addition, we also calculated the C-index. The C-index in the GSE141910 data set used in this study is 0.95, and in the external data set GSE116250(C-index = 0.90), GSE43435(C-index = 0.96), GSE21610(C-index = 0.74), all indicate excellent predictive performance (Fig. 8D). A decision curve means that > 1% to < 98% of the population can follow the nomogram to predict the incidence of DCM (Fig. 8C).

## Discussion

DCM is most common in middle-aged people, and the onset more than 10 years. It is characterized by enlarged left or right ventricle or bilateral ventricles, accompanied by myocardial hypertrophy. Decreased ventricular systolic function, with or without congestive heart failure. Ventricular or atrial arrhythmia is more common. And death can occur at any stage of the disease. At present, dilated cardiomyopathy is diagnosed through exclusion method, that is, excluding other specific causes of cardiac enlargement and cardiac insufficiency, and the diagnosis based on clinical manifestations and auxiliary examinations^1^. It is difficult to prevent DCM due to the unclear mechanism. Therefore, the early diagnosis of DCM will improve the prognosis and quality of life of DCM patients. With the development of genomics, it has been widely used to predict disease susceptibility, drug response and early diagnosis of disease^4^. In this study, 333 differentially expressed genes were identified by analyzing the GSE141910 microarray, and then 197 genes significantly related to DCM were obtained using WGCNA. Among the genes obtained from WGCNA, RGS, FKBP5, STAT4, MYH6, COL9A1 and other genes were confirmed to be associated with DCM progression^17–21^. In addition, ACE-targeted inhibitors have been extensively studied in the treatment of DCM.

We have observed that the expression of NPPA and NPPB are elevated. The DCM patients' ventricular enlargement and reduced myocardial systolic function force the atria and cardiomyocytes to express and release ANP and BNP, thereby exerting diuretic, vasodilatory and immunosuppressive effects. But interestingly, we also found an increase in the expression of ACE and ATGR2, which is opposed to the function of ANP to relax blood vessels. Among them, the overexpression of ATGR2 may be the leading cause that DCM forces the heart to remodel, fibroblasts are the main cell type, and ATGR2 is the main inducer of this cell^22^. In addition, Yan et al. showed that ATGR2 is up-regulated in ventricular myocytes, and its expression is closely related to the phosphorylated protein levels of PKC-α, PKC-β and p70S6 kinase, which promotes the development of dilated cardiomyopathy and heart failure in vivo^23^. There is also evidence that DCM is closely related to immunity. The disorder of the cellular and humoral immune system should be gone hand in hand with the development of DCM. The infiltration of lymphocytes and monocytes and the increased expression of cell adhesion molecules are often found in DCM patients. The production of autoantibodies and the corresponding side effects have also been widely discussed^24–26^. Nakayama et al. confirmed that the increase in myocardial immune activation was positively correlated with poor prognosis of DCM, and CD163-specifically labeled M2 macrophages were closely related to the ventricular remodeling of DCM^27^. The important cause of DCM also includes genetic predisposition. About 20–35% of DCM cases are reported as familial inheritance^28^. Zhao et al. through sequencing revealed that 7 gene mutations, including MYH6, are closely related to the pathogenesis of DCM^18^.

Subsequently, we conducted enrichment analysis of these DCM related genes and explored their potential mechanisms in DCM. Among them, BP and CC are mainly enriched in the extracellular matrix or structure related to cardiac fibrosis, the transport or regulation of different ions, the regulation of renin–angiotensin, and the related immune response and regulation. In MF, in addition to the structural components of the extracellular matrix, there are also G protein-coupled receptors and CXCR chemokine receptors. G protein-coupled receptors are the largest cell membrane surface receptor family in the human body, which are closely related to the occurrence of diseases such as cancer, heart disease, diabetes, and Alzheimer's disease. Among them, autoantibodies to G-protein-coupled β1-adrenergic receptors and M2-cholinergic receptors are specifically expressed in patients with DCM and are involved in the pathological and physiological functions of DCM^29^. Similarly, CXCR chemokine receptors are also a subfamily of G protein-coupled receptors, and this family of receptors is related to cardiac fibrosis. In patients with DCM and end-stage heart failure, there is a close relationship between persistent atrial fibrillation and extracellular remodeling. In atrial fibrosis and atrial fibrillation, the biochemical mediators that play a central role include angiotensin II, CTGF, PDGF and TGF-β. Cytokines and Cytokine Receptors interaction is also enriched in KEGG, which mediates and initiates cell signal transduction. The cytokines exert their biological effects by binding to corresponding cytokine receptors on the cell surface. According to their structure and signal transduction pathway, cytokine receptors were divided into different families or superfamily, such as type I cytokine receptor, type II cytokine receptor, tumor necrosis factor receptor and chemotactic cytokine receptor. As confirmed by Ohtsuka, the increase in serum VEGF and IL-13 may be closely related to the changes in DCM myocardial tissue structure^30^. Chen et al. found that TNF-α gene polymorphism (G-308A) possibly be related to the susceptibility to DCM^31^. These evidence clarified the underlying mechanism of the physiological and pathological development of DCM.

In addition, we conducted a series of analyses to identify 7 hub genes in DCM. Among them, ATGR2 induces cardiac fibroblasts to remodel the DCM heart and promote the development of mental failure^22,23^. While CXCL9, CXCL10, and CXCL11 belong to the chemokine family, CX3CR1, ADORA3 and SAA1 have not been reported in dilated cardiomyopathy, and further research is needed. Finally, we constructed a nomogram for predicting the incidence of DCM based on the expression levels of these 7 genes, and conducted multiple verifications, which not only confirmed its good predictive performance, but also revealed its wide applicability. It can help clinicians and patients diagnose DCM as early as possible, and carry out timely interventions to obtain a better prognosis and improve the quality of life of patients.

## Supplementary Information

Supplementary Table 1.

## Data Availability

All data can be obtained from the corresponding author's office and public databases.
