# Supplementary material for: Development and verification of the nomogram for dilated cardiomyopathy gene diagnosis
Source: Sci Rep. 2022 May 26;12:8908. doi: 10.1038/s41598-022-13135-y (PMC9135684; doi:10.1038/s41598-022-13135-y)
Supplement: Supplementary file 1 — Supplementary Table 1. [file 41598_2022_13135_MOESM1_ESM.docx]

**Supplementary Table 1** Differentially expressed genes in dilated cardiomyopathy.

| ID | Gene Name | logFC | adj.P.Val |
| --- | --- | --- | --- |
| ENSG00000159640 | ACE | 1.959628578 | 6.11E-45 |
| ENSG00000168619 | ADAM18 | 1.80460112 | 2.60E-21 |
| ENSG00000138316 | ADAMTS14 | 1.593597072 | 1.04E-30 |
| ENSG00000158859 | ADAMTS4 | -2.033427645 | 8.79E-41 |
| ENSG00000196616 | ADH1B | -1.515197916 | 6.82E-31 |
| ENSG00000180772 | AGTR2 | 2.272132253 | 1.20E-33 |
| ENSG00000154027 | AK5 | 1.790653367 | 9.76E-50 |
| ENSG00000273259 | AL049839.2 | -2.395557518 | 2.85E-51 |
| ENSG00000179593 | ALOX15B | -2.817148127 | 7.69E-34 |
| ENSG00000135409 | AMHR2 | 1.511489801 | 3.35E-23 |
| ENSG00000235711 | ANKRD34C | 2.159371404 | 1.33E-37 |
| ENSG00000138356 | AOX1 | -2.540758512 | 5.03E-58 |
| ENSG00000198768 | APCDD1L | 3.052113596 | 2.60E-58 |
| ENSG00000231290 | APCDD1L-AS1 | 1.589269837 | 1.07E-27 |
| ENSG00000134817 | APLNR | 1.821600759 | 4.06E-44 |
| ENSG00000118137 | APOA1 | 1.82636409 | 1.39E-25 |
| ENSG00000143595 | AQP10 | 2.291640229 | 1.05E-53 |
| ENSG00000165272 | AQP3 | -1.647734373 | 1.81E-44 |
| ENSG00000171885 | AQP4 | -2.32279594 | 1.76E-29 |
| ENSG00000169126 | ARMC4 | 1.93135641 | 1.03E-43 |
| ENSG00000254636 | ARMS2 | 2.776767084 | 5.11E-55 |
| ENSG00000106819 | ASPN | 2.089154265 | 1.33E-56 |
| ENSG00000132681 | ATP1A4 | 1.675386361 | 6.88E-35 |
| ENSG00000101892 | ATP1B4 | 1.753792428 | 2.84E-14 |
| ENSG00000107518 | ATRNL1 | 1.520805783 | 7.69E-19 |
| ENSG00000101197 | BIRC7 | 1.709658627 | 1.65E-16 |
| ENSG00000197299 | BLM | -1.942638241 | 2.58E-62 |
| ENSG00000153446 | C16orf89 | 1.730384988 | 3.83E-35 |
| ENSG00000180999 | C1orf105 | -2.056842217 | 6.76E-36 |
| ENSG00000145861 | C1QTNF2 | 1.709485958 | 8.35E-58 |
| ENSG00000163145 | C1QTNF7 | 1.717865633 | 3.39E-61 |
| ENSG00000119147 | C2orf40 | 1.601131946 | 1.18E-39 |
| ENSG00000221972 | C3orf36 | -1.934830681 | 4.19E-35 |
| ENSG00000178722 | C5orf64 | -1.610592657 | 3.21E-23 |
| ENSG00000118298 | CA14 | -1.536251127 | 4.19E-23 |
| ENSG00000077274 | CAPN6 | 2.634305988 | 1.58E-37 |
| ENSG00000106178 | CCL24 | 1.617091139 | 3.96E-19 |
| ENSG00000126353 | CCR7 | 1.527328096 | 1.76E-20 |
| ENSG00000177575 | CD163 | -2.472235235 | 3.00E-73 |
| ENSG00000204936 | CD177 | -2.134143837 | 1.02E-20 |
| ENSG00000158481 | CD1C | 2.172162771 | 8.71E-39 |
| ENSG00000158488 | CD1E | 1.598694392 | 9.89E-21 |
| ENSG00000116824 | CD2 | 1.576892211 | 4.27E-29 |
| ENSG00000167286 | CD3D | 1.710206283 | 6.09E-29 |
| ENSG00000198851 | CD3E | 1.65675959 | 5.95E-33 |
| ENSG00000160654 | CD3G | 1.531750639 | 1.36E-24 |
| ENSG00000013725 | CD6 | 1.628161608 | 1.52E-28 |
| ENSG00000170956 | CEACAM3 | 1.534107608 | 6.38E-20 |
| ENSG00000115163 | CENPA | 2.368579765 | 8.81E-28 |
| ENSG00000223802 | CERS1 | 1.722325566 | 2.02E-23 |
| ENSG00000001626 | CFTR | -2.105334783 | 2.90E-32 |
| ENSG00000116254 | CHD5 | 1.761526265 | 8.57E-32 |
| ENSG00000080644 | CHRNA3 | 2.646860187 | 4.95E-41 |
| ENSG00000092009 | CMA1 | 2.448562458 | 9.19E-34 |
| ENSG00000140932 | CMTM2 | 1.628082777 | 7.47E-20 |
| ENSG00000113805 | CNTN3 | -1.703439422 | 4.63E-26 |
| ENSG00000123500 | COL10A1 | 2.500317831 | 3.09E-35 |
| ENSG00000187955 | COL14A1 | 1.6380225 | 8.20E-42 |
| ENSG00000169436 | COL22A1 | 3.433498319 | 3.35E-66 |
| ENSG00000112280 | COL9A1 | 1.949547946 | 1.98E-42 |
| ENSG00000105664 | COMP | 2.099921084 | 2.23E-17 |
| ENSG00000175874 | CREG2 | 1.788453964 | 1.88E-29 |
| ENSG00000121005 | CRISPLD1 | 2.448477608 | 5.56E-47 |
| ENSG00000249201 | CTD-3080P12.3 | 1.600908048 | 6.70E-16 |
| ENSG00000100448 | CTSG | 2.007037301 | 2.94E-30 |
| ENSG00000172543 | CTSW | 1.954005301 | 1.20E-32 |
| ENSG00000168329 | CX3CR1 | 2.004346247 | 2.53E-37 |
| ENSG00000169245 | CXCL10 | 2.632187873 | 7.00E-33 |
| ENSG00000169248 | CXCL11 | 2.191793916 | 1.05E-24 |
| ENSG00000138755 | CXCL9 | 1.791900765 | 4.33E-18 |
| ENSG00000140459 | CYP11A1 | 1.650276681 | 6.97E-46 |
| ENSG00000142973 | CYP4B1 | -2.520794783 | 9.58E-49 |
| ENSG00000186529 | CYP4F3 | 1.876457801 | 6.32E-27 |
| ENSG00000186160 | CYP4Z1 | -2.223800578 | 7.26E-47 |
| ENSG00000205795 | CYS1 | 1.703538608 | 1.17E-61 |
| ENSG00000225756 | DBH-AS1 | 1.704087886 | 2.27E-35 |
| ENSG00000184544 | DHRS7C | -1.840217247 | 4.60E-17 |
| ENSG00000124721 | DNAH8 | 1.570480428 | 1.98E-25 |
| ENSG00000198692 | EIF1AY | 1.727328331 | 0.003369953 |
| ENSG00000124882 | EREG | -2.064495349 | 5.96E-19 |
| ENSG00000164283 | ESM1 | 2.158170434 | 4.60E-29 |
| ENSG00000164089 | ETNPPL | -1.546352024 | 1.96E-26 |
| ENSG00000205436 | EXOC3L4 | 1.705221783 | 1.83E-21 |
| ENSG00000130054 | FAM155B | -1.551330042 | 1.63E-24 |
| ENSG00000196666 | FAM180B | 2.034386783 | 1.94E-35 |
| ENSG00000078098 | FAP | 1.577404928 | 8.36E-30 |
| ENSG00000147378 | FATE1 | 2.006847627 | 3.98E-32 |
| ENSG00000179639 | FCER1A | 2.734235958 | 6.73E-53 |
| ENSG00000162747 | FCGR3B | 1.972386337 | 1.43E-27 |
| ENSG00000085265 | FCN1 | 1.555875078 | 2.18E-21 |
| ENSG00000142748 | FCN3 | -2.430349777 | 8.51E-59 |
| ENSG00000160856 | FCRL3 | 1.778432813 | 6.24E-27 |
| ENSG00000181036 | FCRL6 | 1.580650759 | 1.50E-24 |
| ENSG00000070193 | FGF10 | -1.926848578 | 3.74E-22 |
| ENSG00000233485 | FHAD1-AS1 | 3.561098554 | 1.68E-78 |
| ENSG00000096060 | FKBP5 | -2.130015657 | 2.80E-55 |
| ENSG00000122176 | FMOD | 1.594250795 | 2.62E-37 |
| ENSG00000164694 | FNDC1 | 2.981556699 | 1.97E-85 |
| ENSG00000164946 | FREM1 | 1.820944976 | 1.11E-75 |
| ENSG00000162998 | FRZB | 2.140647217 | 9.72E-80 |
| ENSG00000131386 | GALNT15 | -2.131049892 | 1.97E-58 |
| ENSG00000172020 | GAP43 | 1.772267458 | 3.72E-22 |
| ENSG00000156466 | GDF6 | 1.978032976 | 1.80E-41 |
| ENSG00000248587 | GDNF-AS1 | 1.755062181 | 2.44E-37 |
| ENSG00000146013 | GFRA3 | 1.547079524 | 5.33E-29 |
| ENSG00000174332 | GLIS1 | 2.246601259 | 6.83E-36 |
| ENSG00000205835 | GMNC | -1.934517759 | 4.66E-27 |
| ENSG00000124713 | GNMT | -2.551454464 | 1.15E-49 |
| ENSG00000119714 | GPR68 | 1.843546187 | 1.36E-27 |
| ENSG00000139572 | GPR84 | -1.579169813 | 1.01E-19 |
| ENSG00000169181 | GSG1L | 1.520943524 | 6.18E-11 |
| ENSG00000134201 | GSTM5 | 1.672163723 | 1.07E-40 |
| ENSG00000145649 | GZMA | 1.840364241 | 1.13E-38 |
| ENSG00000100453 | GZMB | 2.181143018 | 2.23E-36 |
| ENSG00000100450 | GZMH | 2.705182464 | 1.57E-49 |
| ENSG00000162882 | HAAO | 1.56030162 | 4.28E-41 |
| ENSG00000145681 | HAPLN1 | 2.777067964 | 8.79E-41 |
| ENSG00000206172 | HBA1 | 3.272490693 | 3.31E-30 |
| ENSG00000188536 | HBA2 | 3.551896127 | 1.04E-29 |
| ENSG00000244734 | HBB | 3.33099409 | 5.82E-39 |
| ENSG00000140287 | HDC | 1.637229006 | 2.10E-29 |
| ENSG00000134240 | HMGCS2 | -1.981258096 | 4.74E-15 |
| ENSG00000229221 | HNRNPA1P66 | 2.14770209 | 2.32E-31 |
| ENSG00000171476 | HOPX | -2.041821952 | 1.96E-30 |
| ENSG00000135116 | HRK | 2.070924139 | 1.60E-37 |
| ENSG00000196684 | HSH2D | 1.952168337 | 1.52E-39 |
| ENSG00000102468 | HTR2A | 1.652121337 | 4.96E-23 |
| ENSG00000106302 | HYAL4 | 1.523549892 | 2.08E-26 |
| ENSG00000211890 | IGHA2 | 1.520224392 | 1.56E-09 |
| ENSG00000211892 | IGHG4 | 1.732880831 | 9.38E-09 |
| ENSG00000211679 | IGLC3 | 1.515594223 | 1.82E-11 |
| ENSG00000136634 | IL10 | -2.284933506 | 2.94E-41 |
| ENSG00000172458 | IL17D | 1.529695952 | 6.06E-44 |
| ENSG00000056736 | IL17RB | -1.686514548 | 2.83E-36 |
| ENSG00000115604 | IL18R1 | -1.586176855 | 5.14E-47 |
| ENSG00000115590 | IL1R2 | -2.249817024 | 1.20E-37 |
| ENSG00000115602 | IL1RL1 | -4.131266988 | 9.72E-80 |
| ENSG00000100385 | IL2RB | 1.566518066 | 2.04E-34 |
| ENSG00000157368 | IL34 | 1.554477133 | 1.46E-39 |
| ENSG00000129009 | ISLR | 1.825781867 | 9.95E-44 |
| ENSG00000005844 | ITGAL | 1.669021271 | 8.22E-42 |
| ENSG00000182132 | KCNIP1 | 2.152892416 | 3.73E-43 |
| ENSG00000124780 | KCNK17 | 1.759569711 | 8.68E-25 |
| ENSG00000109265 | KIAA1211 | 2.363124367 | 1.33E-30 |
| ENSG00000149633 | KIAA1755 | 1.569298608 | 6.63E-47 |
| ENSG00000162873 | KLHDC8A | 1.678434301 | 1.55E-30 |
| ENSG00000111796 | KLRB1 | 1.623632367 | 9.96E-29 |
| ENSG00000159166 | LAD1 | -1.923526072 | 3.09E-35 |
| ENSG00000125869 | LAMP5 | 2.547793398 | 4.13E-58 |
| ENSG00000129988 | LBP | -2.082202175 | 2.46E-17 |
| ENSG00000187922 | LCN10 | -2.954448699 | 5.02E-51 |
| ENSG00000184925 | LCN12 | 1.625949416 | 1.44E-27 |
| ENSG00000177984 | LCN15 | -2.2652165 | 3.07E-28 |
| ENSG00000267206 | LCN6 | -2.519730127 | 9.32E-51 |
| ENSG00000143768 | LEFTY2 | 2.807439139 | 9.26E-63 |
| ENSG00000168481 | LGI3 | -1.963037873 | 8.87E-26 |
| ENSG00000138039 | LHCGR | 1.746894693 | 5.13E-20 |
| ENSG00000229246 | LINC00377 | 2.223171392 | 4.05E-43 |
| ENSG00000179136 | LINC00670 | 1.69397712 | 8.90E-44 |
| ENSG00000231298 | LINC00704 | -1.68216791 | 5.45E-35 |
| ENSG00000245164 | LINC00861 | 1.902643994 | 6.37E-36 |
| ENSG00000249816 | LINC00964 | -1.738092536 | 4.14E-31 |
| ENSG00000236819 | LINC01563 | 1.740569145 | 5.78E-34 |
| ENSG00000238755 | LINC02006 | -1.884478783 | 1.87E-29 |
| ENSG00000260186 | LINC02137 | -2.41781012 | 2.33E-56 |
| ENSG00000258476 | LINC02207 | -1.672179476 | 5.33E-24 |
| ENSG00000271538 | LINC02427 | 1.750132512 | 1.04E-26 |
| ENSG00000260807 | LMF1 | 1.53233559 | 3.34E-36 |
| ENSG00000223764 | LOC100130417 | 2.298156807 | 1.68E-43 |
| ENSG00000233968 | LOC101928834 | 1.984043633 | 4.94E-39 |
| ENSG00000233098 | LOC105369205 | 2.392461434 | 5.52E-61 |
| ENSG00000101425 | LOC149684 | 1.610356795 | 3.17E-26 |
| ENSG00000230102 | LOC285389 | 1.572781337 | 1.88E-36 |
| ENSG00000248713 | LOC285556 | -2.141729373 | 1.05E-17 |
| ENSG00000172061 | LRRC15 | 1.752609765 | 3.51E-26 |
| ENSG00000128606 | LRRC17 | 1.556262422 | 2.71E-46 |
| ENSG00000183908 | LRRC55 | 2.365508988 | 1.42E-36 |
| ENSG00000227507 | LTB | 1.935300717 | 4.31E-26 |
| ENSG00000139329 | LUM | 1.944741964 | 2.68E-62 |
| ENSG00000122224 | LY9 | 1.578388108 | 6.98E-24 |
| ENSG00000150551 | LYPD1 | 2.707971265 | 2.53E-41 |
| ENSG00000133800 | LYVE1 | -1.89795991 | 5.15E-54 |
| ENSG00000019169 | MARCO | -1.668877048 | 1.58E-12 |
| ENSG00000183019 | MCEMP1 | -2.177028337 | 3.91E-29 |
| ENSG00000110492 | MDK | 2.006628295 | 9.37E-40 |
| ENSG00000228109 | MELTF-AS1 | 1.720984548 | 3.24E-48 |
| ENSG00000170439 | METTL7B | -1.819107078 | 1.04E-33 |
| ENSG00000166482 | MFAP4 | 1.650729 | 1.58E-66 |
| ENSG00000008394 | MGST1 | -1.71141162 | 4.48E-33 |
| ENSG00000106624 | MIR4649 | 1.661965392 | 1.32E-39 |
| ENSG00000130592 | MIR7847 | 1.772508759 | 9.70E-48 |
| ENSG00000225526 | MKRN2OS | 2.615130012 | 4.58E-29 |
| ENSG00000156738 | MS4A1 | 1.612034741 | 1.88E-19 |
| ENSG00000205362 | MT1A | -1.503259241 | 1.18E-10 |
| ENSG00000101825 | MXRA5 | 2.623624446 | 5.15E-54 |
| ENSG00000197616 | MYH6 | -2.756116904 | 9.30E-65 |
| ENSG00000168530 | MYL1 | 1.623850976 | 2.07E-18 |
| ENSG00000034971 | MYOC | 1.660083916 | 1.79E-29 |
| ENSG00000131401 | NAPSB | 1.611239048 | 2.92E-26 |
| ENSG00000124479 | NDP | 1.850347855 | 1.19E-31 |
| ENSG00000237928 | NFIA-AS2 | 1.93367244 | 6.55E-42 |
| ENSG00000066248 | NGEF | 1.517682837 | 3.43E-19 |
| ENSG00000105374 | NKG7 | 2.294541422 | 1.39E-37 |
| ENSG00000086288 | NME8 | 1.5606045 | 8.08E-25 |
| ENSG00000171658 | NMRAL2P | -1.79962112 | 8.97E-37 |
| ENSG00000175206 | NPPA | 2.194547048 | 9.90E-25 |
| ENSG00000120937 | NPPB | 2.086643554 | 1.94E-15 |
| ENSG00000106236 | NPTX2 | -2.503962024 | 3.98E-58 |
| ENSG00000123572 | NRK | 1.678027512 | 2.37E-53 |
| ENSG00000110076 | NRXN2 | 1.717786199 | 4.53E-33 |
| ENSG00000182667 | NTM | 1.696248536 | 3.45E-55 |
| ENSG00000135114 | OASL | 1.826924434 | 1.10E-42 |
| ENSG00000197444 | OGDHL | 1.791872729 | 1.07E-39 |
| ENSG00000106809 | OGN | 1.862707651 | 1.62E-43 |
| ENSG00000152463 | OLAH | -2.277953801 | 1.09E-41 |
| ENSG00000183715 | OPCML | 1.657874512 | 5.09E-32 |
| ENSG00000187950 | OVCH1 | -1.783609066 | 6.23E-33 |
| ENSG00000177359 | OVOS2 | -2.218001151 | 3.63E-22 |
| ENSG00000162881 | OXER1 | 1.740833627 | 2.78E-43 |
| ENSG00000090530 | P3H2 | 1.570716482 | 7.81E-33 |
| ENSG00000175426 | PCSK1 | -1.642920916 | 1.70E-20 |
| ENSG00000181195 | PENK | 3.306105687 | 5.20E-60 |
| ENSG00000139289 | PHLDA1 | 1.550435867 | 2.50E-53 |
| ENSG00000137558 | PI15 | -2.819597018 | 2.83E-36 |
| ENSG00000164530 | PI16 | 1.915591639 | 3.32E-41 |
| ENSG00000188257 | PLA2G2A | -2.478716675 | 2.63E-35 |
| ENSG00000168907 | PLA2G4F | -1.862948964 | 2.65E-21 |
| ENSG00000086717 | PPEF1 | -2.412339422 | 1.67E-43 |
| ENSG00000156475 | PPP2R2B | 2.071375855 | 1.32E-51 |
| ENSG00000174899 | PQLC2L | -1.519515825 | 4.15E-38 |
| ENSG00000180644 | PRF1 | 1.854466783 | 6.07E-39 |
| ENSG00000115592 | PRKAG3 | 2.028015241 | 8.06E-43 |
| ENSG00000007062 | PROM1 | 1.55721756 | 1.02E-44 |
| ENSG00000146250 | PRSS35 | 1.659384988 | 3.20E-27 |
| ENSG00000105894 | PTN | 1.503342825 | 4.74E-54 |
| ENSG00000118849 | RARRES1 | -2.07053909 | 1.54E-37 |
| ENSG00000165105 | RASEF | 1.701460705 | 7.05E-28 |
| ENSG00000128045 | RASL11B | 1.537933181 | 1.49E-44 |
| ENSG00000250295 | RDH10-AS1 | -1.50464056 | 8.66E-34 |
| ENSG00000117152 | RGS4 | 1.836959789 | 3.31E-37 |
| ENSG00000141314 | RHBDL3 | -1.763905151 | 2.55E-26 |
| ENSG00000169385 | RNASE2 | -3.155700898 | 7.27E-63 |
| ENSG00000129824 | RPS4Y1 | 1.695972199 | 0.00374951 |
| ENSG00000198838 | RYR3 | 1.823578753 | 3.51E-38 |
| ENSG00000180739 | S1PR5 | 1.70300038 | 1.07E-25 |
| ENSG00000173432 | SAA1 | -2.37427391 | 8.18E-12 |
| ENSG00000187634 | SAMD11 | 1.931878988 | 4.40E-24 |
| ENSG00000164483 | SAMD3 | 1.856192892 | 1.07E-40 |
| ENSG00000123453 | SARDH | 1.548492886 | 1.17E-36 |
| ENSG00000079689 | SCGN | -2.392810096 | 1.39E-35 |
| ENSG00000175356 | SCUBE2 | 2.150340163 | 4.69E-67 |
| ENSG00000196136 | SERPINA3 | -2.138708199 | 4.17E-25 |
| ENSG00000100095 | SEZ6L | 3.515280855 | 6.67E-76 |
| ENSG00000106483 | SFRP4 | 3.442255102 | 1.11E-75 |
| ENSG00000163082 | SGPP2 | -2.353709163 | 4.73E-39 |
| ENSG00000198574 | SH2D1B | 1.609783277 | 4.01E-26 |
| ENSG00000180730 | SHISA2 | 1.881610633 | 1.25E-35 |
| ENSG00000171101 | SIGLEC17P | 1.726294133 | 1.40E-32 |
| ENSG00000129450 | SIGLEC9 | -1.610438771 | 1.13E-48 |
| ENSG00000232044 | SILC1 | -1.909481265 | 6.08E-24 |
| ENSG00000026751 | SLAMF7 | 1.897396078 | 5.42E-31 |
| ENSG00000018280 | SLC11A1 | -1.748260518 | 5.97E-36 |
| ENSG00000165449 | SLC16A9 | 1.782736657 | 4.72E-51 |
| ENSG00000162383 | SLC1A7 | 2.859543217 | 2.09E-49 |
| ENSG00000182902 | SLC25A18 | -1.622896759 | 7.77E-32 |
| ENSG00000166558 | SLC38A8 | -1.57332753 | 2.27E-12 |
| ENSG00000108576 | SLC6A4 | 1.679692386 | 3.38E-21 |
| ENSG00000101187 | SLCO4A1 | -2.362767765 | 2.46E-57 |
| ENSG00000173930 | SLCO4C1 | 1.51854788 | 3.73E-25 |
| ENSG00000112562 | SMOC2 | 1.830076777 | 5.84E-92 |
| ENSG00000179954 | SSC5D | 2.040334006 | 4.32E-65 |
| ENSG00000162009 | SSTR5 | -1.612410223 | 8.05E-09 |
| ENSG00000136011 | STAB2 | 1.679112506 | 7.50E-22 |
| ENSG00000141750 | STAC2 | -1.95876556 | 3.51E-29 |
| ENSG00000138378 | STAT4 | 1.75941906 | 1.04E-34 |
| ENSG00000173597 | SULT1B1 | -2.030982675 | 9.31E-44 |
| ENSG00000157152 | SYN2 | -1.969958602 | 2.11E-36 |
| ENSG00000147041 | SYTL5 | 1.735473139 | 1.11E-15 |
| ENSG00000211689 | TARP | 1.556282494 | 1.66E-25 |
| ENSG00000175463 | TBC1D10C | 1.506487163 | 8.62E-24 |
| ENSG00000266733 | TBC1D29 | -1.671600693 | 1.61E-22 |
| ENSG00000073861 | TBX21 | 1.694437042 | 8.20E-33 |
| ENSG00000261787 | TCF24 | -1.975356464 | 9.40E-21 |
| ENSG00000156414 | TDRD9 | -1.66762138 | 1.12E-50 |
| ENSG00000149256 | TENM4 | 1.550780313 | 1.41E-37 |
| ENSG00000232480 | TGFB2-AS1 | 2.126061928 | 1.70E-34 |
| ENSG00000154096 | THY1 | 1.598235958 | 7.38E-19 |
| ENSG00000095587 | TLL2 | 1.68036462 | 3.39E-61 |
| ENSG00000144339 | TMEFF2 | -1.660614687 | 5.36E-18 |
| ENSG00000183160 | TMEM119 | 1.779334078 | 6.75E-29 |
| ENSG00000166448 | TMEM130 | 2.177306018 | 1.50E-27 |
| ENSG00000164484 | TMEM200A | 1.652283892 | 3.45E-20 |
| ENSG00000121933 | TMIGD3 | -1.52366362 | 3.19E-34 |
| ENSG00000186827 | TNFRSF4 | 1.52836006 | 6.37E-12 |
| ENSG00000000005 | TNMD | 2.550095205 | 1.32E-36 |
| ENSG00000159173 | TNNI1 | 1.866222464 | 3.52E-17 |
| ENSG00000211829 | TRDC | 1.804613151 | 1.21E-26 |
| ENSG00000227191 | TRGC2 | 1.575785139 | 4.76E-24 |
| ENSG00000198033 | TUBA3C | -2.596405398 | 1.53E-45 |
| ENSG00000075886 | TUBA3D | -2.821248771 | 2.62E-59 |
| ENSG00000152086 | TUBA3E | -2.852473337 | 2.68E-61 |
| ENSG00000144406 | UNC80 | 2.597658747 | 2.20E-43 |
| ENSG00000114374 | USP9Y | 1.520914753 | 0.00782116 |
| ENSG00000170162 | VGLL2 | 1.60881897 | 1.94E-13 |
| ENSG00000155659 | VSIG4 | -1.95064153 | 1.32E-63 |
| ENSG00000196632 | WNK3 | -1.894899325 | 1.08E-53 |
| ENSG00000143816 | WNT9A | 2.011213584 | 9.36E-33 |
| ENSG00000124343 | XG | 1.941042139 | 2.29E-28 |
| ENSG00000115085 | ZAP70 | 1.556453229 | 8.10E-29 |
| ENSG00000124256 | ZBP1 | 1.522720867 | 2.96E-29 |
| ENSG00000179300 | ZCCHC5 | 2.132839886 | 9.47E-50 |
| ENSG00000138311 | ZNF365 | 2.070012928 | 2.19E-39 |
| ENSG00000090920 | ENSG00000090920 | -1.852930096 | 4.03E-22 |
| ENSG00000161570 | ENSG00000161570 | 2.239918265 | 6.47E-47 |
| ENSG00000224842 | ENSG00000224842 | 1.617854072 | 2.70E-34 |
| ENSG00000232110 | ENSG00000232110 | 1.820267199 | 1.11E-31 |
| ENSG00000249631 | ENSG00000249631 | -1.518794699 | 4.06E-34 |
| ENSG00000250971 | ENSG00000250971 | 1.505200777 | 3.18E-21 |
| ENSG00000255146 | ENSG00000255146 | 1.548426337 | 3.77E-27 |
| ENSG00000257181 | ENSG00000257181 | -1.51682306 | 1.33E-22 |
| ENSG00000260484 | ENSG00000260484 | -1.626798645 | 9.15E-22 |
| ENSG00000263257 | ENSG00000263257 | -1.930948693 | 2.37E-33 |
| ENSG00000265356 | ENSG00000265356 | -1.70206503 | 3.73E-16 |
| ENSG00000265542 | ENSG00000265542 | -2.419235831 | 2.58E-62 |
| ENSG00000266743 | ENSG00000266743 | 1.97461388 | 1.26E-30 |
| ENSG00000267577 | ENSG00000267577 | 2.178948114 | 1.07E-48 |
| ENSG00000267653 | ENSG00000267653 | -2.49715659 | 4.98E-41 |
| ENSG00000271984 | ENSG00000271984 | -2.372868831 | 3.32E-29 |
| ENSG00000272268 | ENSG00000272268 | -1.706532741 | 5.54E-19 |
| ENSG00000272327 | ENSG00000272327 | 2.02745144 | 1.59E-34 |
| ENSG00000272473 | ENSG00000272473 | -1.719786699 | 1.42E-23 |
| ENSG00000272970 | ENSG00000272970 | 1.682611536 | 1.70E-29 |
| ENSG00000273403 | ENSG00000273403 | 1.657004657 | 1.43E-29 |
